# Plant–Microbe Interaction: Aboveground to Belowground, from the Good to the Bad

**DOI:** 10.3390/ijms221910388

**Published:** 2021-09-27

**Authors:** Kalaivani Nadarajah, Nur Sabrina Natasha Abdul Rahman

**Affiliations:** Department of Biological Sciences and Biotechnology, Faculty of Science and Technology, Universiti Kebangsaan Malaysia, Bangi 43600, Malaysia; nursabrinatasha@gmail.com

**Keywords:** microbiome, aboveground, belowground, associations, niche communities

## Abstract

Soil health and fertility issues are constantly addressed in the agricultural industry. Through the continuous and prolonged use of chemical heavy agricultural systems, most agricultural lands have been impacted, resulting in plateaued or reduced productivity. As such, to invigorate the agricultural industry, we would have to resort to alternative practices that will restore soil health and fertility. Therefore, in recent decades, studies have been directed towards taking a Magellan voyage of the soil rhizosphere region, to identify the diversity, density, and microbial population structure of the soil, and predict possible ways to restore soil health. Microbes that inhabit this region possess niche functions, such as the stimulation or promotion of plant growth, disease suppression, management of toxicity, and the cycling and utilization of nutrients. Therefore, studies should be conducted to identify microbes or groups of organisms that have assigned niche functions. Based on the above, this article reviews the aboveground and below-ground microbiomes, their roles in plant immunity, physiological functions, and challenges and tools available in studying these organisms. The information collected over the years may contribute toward future applications, and in designing sustainable agriculture.

## 1. Introduction

Plants are colonized aboveground and belowground by mutualistic and parasitic organisms. These organisms can be categorized into groups based on areas of colonization; for instance, microorganisms that colonize the external parts of the plant are generally known as epiphytes, while those that colonize the inside of the plants are endophytes. Furthermore, there are phyllosphere organisms that colonize the leaf surface; and the most abundant group of them would be the rhizosphere inhabitants, which colonize regions closest to the root system [1,2,3]. This region is teaming with microbes, which are attracted to the root systems due to their exudates. The exudates depend on the developmental stages and physiological statuses of the plants [4,5]. Although the recruitment of microbes to the root region may be a consequence of plant exudation, the microorganisms that colonize this region have diverse roles in supporting plant growth, development, and inhibition of host pathogens. This implies interdependency between the host and microbes in the aboveground and belowground interactions [6,7].

The microbial diversification, speciation, structural complexity, and interactions that surround the root systems make it essential to understand the microbial population as well as the root architecture, to have a clear view on how these interactome associate [8]. Due to the high levels of interaction between the plant and microbes, these components are observed as holobionts or metaorganisms [9,10]. In addition to the intertwining plant and microbe associations (plant-microbe–plant), there is the microbe–microbe and microbe–soil association. The complexity of the microbes in soil is not just circumnavigated by the plant, but by the environment and other constituents in the soil. The physicochemical and biological components of the soil largely influence the microbiome. For instance, climate change and its effect on agriculture, such as drought and flooding, severely impact soil microbiomes. Furthermore, physical changes in temperature, pH, oxygen level, and soil structure also affect its inhabitants [11]. In addition, the chemical compounds derived through the cycling of materials in the soil or from agricultural practices also affects soil biology. Microbes that adapt to a particular stress condition might be beneficial to plants, since beneficial microbes are shown to increase soil health and fertility. This is not inclusive of the role played by macro- and microorganisms belowground and aboveground in influencing the plant-microbe interaction (such as animal grazing, etc.) [5].

Beneficial microorganisms can be inoculated in soil or used as input to improve agricultural practices. Microbial inoculants are administered to the plant or the soil to boost crop productivity and health, and mitigate the negative effects of agrochemicals. It is a viable alternative to chemical treatment and is capable of promoting plant development, controlling pests and diseases, and stabilizing soil structure. These inputs may be employed as biocontrol agents, biopesticides, bioherbicides, and biofertilizers. Over the last few decades, significant developments have been made in manufacturing, marketing, and use of inoculants [12]. Nowadays, the use of inoculants is more widespread, owing to the availability of excellent and multifunctional strains in the market, improving yield at a lower cost than synthetic fertilizers. Rhizobia are the most extensively utilized microbes as inoculants [13]. The legume–rhizobia symbiosis influences the mechanism of biological nitrogen fixation (BNF), which satisfies the plant’s N needs [12]. Plant growth-promoting bacteria (PGPB) can support a plant in a range of areas on its own or in combination with other factors. PGPB influences plants through phytohormones and siderophores synthesis, phosphate solubilization, and elicitation of a plant’s internal defense against biotic and abiotic stressors [14,15]. Various microorganisms are increasingly being employed in agriculture for ecological pest and disease management [16].

The recent surge in new technologies in genome studies has enabled us to further characterize the microbial diversity, genome, and proteome of microorganisms living in association in soil or on plants. DNA/RNA, genome analysis, transcriptome, proteome, metagenome, and all other omics-based technologies have provided a means to dissect beneficial and non-beneficial plant-microbe interactions at depths and speeds that were not possible decades ago. These technologies enable us to understand the dynamics belowground and aboveground, to further utilize this information to improve growth, yield, and disease reduction [17]. If we are able to decipher the factors responsible for the establishment of the microbial communities in the rhizosphere, we will be able to utilize this information in designing sustainable ecosystems that are beneficial, stable, and productive for the long haul [18,19]. Despite the fact that the ecosystem was sustainable prior to interference, this approach restores the environment to its original state before human intervention. Hence, given the above background, this current review focuses on the aboveground and belowground microbial interactions, the development of diseases and emerging threats, the beneficial uses of microbes, and the available new tools to study them at greater depth (see Supplementary Figure S1 for the methodology of Systematic Review for Plant Microbe Interactions).

## 2. The Two-Phase Microbial Communities

### 2.1. Aboveground Microbes

Endophytic and epiphytic groups of microorganisms colonize and inhabit plant tissues, such as leaves and flowers [20]. The phyllosphere organisms (those that are on external plant surfaces) will generally be influenced by the environment and may be commensal-like organisms, or organisms that can cause disease. The phyllosphere is a severe and unstable environment with oligotrophy-like features, such as nutritional restriction in carbon and nitrogen, as well as numerous, highly variable physicochemical limitations (light penetration, UV radiation, temperature, desiccation) [21]. Microbial adjustment to the phyllosphere environment appears to be dependent on a number of factors linked to a range of physicochemical and biotic limitations, such as exposure to air, water, soil, animal, or insect borne microbes [1,22,23]. Meanwhile endophytic organisms systematically obtain their microbial nutrients through the xylem and aerial tissue, such as fruits and flowers [22,24]. The distribution of the endophytes within the plant tissue will largely depend on the nutritional source within the organ to support the growth and development of the endophytes. There will be observable differences in the genera between endophytes and the phyllosphere inhabitants [25]. For example, in a study conducted on tomato plants, an *Acinetobacter* dominant community was reported in stems and leaves, while tissues of stems and leaves were colonized by *Xanthomonas*, *Rhizobium*, *Methylobacterium*, *Sphingomonas*, and *Pseudomonas* [2]. When the tomato was compared to other host plants, *Bacillus* and *Pantoea* dominated the lettuce phyllosphere. In potato phyllosphere, *Devosia, Dyadobacter*, and *Pedobacter* were dominant, while *Pseudomonas* dominated the spinach phyllosphere [2]. In another study conducted on maize, the phyllosphere was dominated by *Sphingomonas* and *Methylobacteria* [26]. From this observation, we can conclude that the microbes dominating a particular plant depends on the host tissue, geographical location, tissue nutrient content, and the physicochemical characteristics of the soil [27]. While endophytes in tomatoes are diverse, *Acinetobacter*, *Enterobacter*, *Pseudomonas,* and *Pantoea* were identified as the most dominant, with varying densities in different vegetative tissues. *Enterobacter*, *Pseudomonas,* and *Pantoea* were also commonly found in other host plants [2,28]. However, in grapes, the phyllosphere was inhabited by *Pseudomonas*, *Sphingomonas*, *Frigoribacterium*, *Curtobacterium*, *Bacillus*, *Enterobacter*, *Acinetobacter*, *Erwinia*, *Citrobacter*, *Pantoea*, and *Methylobacterium,* while the endophytes were dominated by *Ralstonia*, *Burkholderia*, *Pseudomonas*, *Staphylococcus*, *Mesorhizobium*, *Propionibacterium*, *Dyella*, and *Bacillus* [2,29]. Aleklett et al. [30] identified *Pseudomonas* and *Enterobacteriaceae* taxa on apple flowers. Pseudomonads were the most abundant genus in floral organs of several fruits, such as apples, grapefruits, and pumpkins.

Resistance and tolerance responses towards antibacterial and immunological chemicals generated by plant tissues, as well as competing microbes, can be developed by epiphytic microorganisms [31]. In the phyllosphere of tobacco, epiphytic bacteria with enzymes, which degrade N-acylhomoserine lactone (AHL) and quorum-sensing signals, have been discovered; thus, it was proposed that signaling circuits may be associated with the formation of complex epiphyllic microbial communities [32]. Epiphytic microbes could also evolve pathways for aggregation or exopolysaccharide production, to increase adherence or resistance to desiccation [33]. Epiphytic organisms could also produce and release phyto-hormonal chemicals, such as indole-3-acetic acid (IAA), by stimulating the loosening of plant cell walls and releasing saccharides from plant cell walls [34]. In conclusion, the relationship between the plant host and aboveground microorganisms is host dependent and largely influenced by the environment and signaling circuits associated to the microbial communities. These organisms may provide beneficial or detrimental relationships with the host, resulting in either enhanced growth and defense or the elicitation of disease and losses, respectively [25,35].

### 2.2. Microbes from Belowground

The rhizosphere is a highly ubiquitous region, with plant exudates, which recruits microbes, creating a microbial reservoir [8]. The microorganisms in the rhizosphere constantly interact with one another, resulting in commensalism, parasitism, amensalism, saprophytism, and symbiotic associations. These organisms affect the aboveground activities and are part of the bulk soil species. Plants and the environment of the soil determine the soil microbial communities [36]. However, nothing is known about how the rhizosphere composition is chosen from bulk soil. Two mechanisms might explain the population—the neutral or niche-based mechanism. The neutral mechanism is based on the fact that most organisms are able to exploit most soil niches and, therefore, are limited by the distance among plants, recruitment parameters, and hampered dispersal [37]. However, for the niche-based mechanism, environmental changes alter the microbial communities [38]. Plants have the tendency to recruit microorganisms to the rhizosphere that will assist with biological functions, such as nutrient uptake, growth, and development. The example seen in cereals is the niche community, where richness and microbial abundance is strongly dependent on the ecological space of the rhizosphere [39]. Similarly, the orchid microflora studies, starting from seed germination through establishment, reproduction, and survival of orchids are heavily reliant on orchid mycorrhizal fungi (OMF). Corollary, changes in OMF composition and abundance can have a substantial influence on dispersion and fitness of orchids [40], providing another example of a niche-based mechanism.

The recruitment agent, exudates/mucilages from the roots, release amino acids, cutin monomers, flavonoids, hormones, organic acids, polyphenols, sugars, and nutrients that are involved in moderating the plant-microbe interactions and microbial gene expression [23,41,42]. These chemicals act as signals; and are deployed to initiate the microbial colonization of roots. While some of these compounds are elicited to enhance plant growth and development, secondary metabolites, such as benzoxazinoids in maize roots, are produced to specifically inhibit *Actinobacteria* and *Proteobacteria* [8,43]. The recruitment of microbiota to the root is mobilized when the machinery involved in biofilm formation, chemotaxis, detoxification, mobility, polysaccharide degradation, and secondary metabolism is switched on [44]. The microbiome begins to expand, establishes niches, and recruits additional microbes through a cross-feeding approach, resulting in new niche groups being developed within the population [45]. Once the microbial population establishes a community around the root, the plant’s exudate shifts its focus toward enhancing the formation of biofilms around the roots [46].

Besides the variations that may be observed between plant genera, the differences in variety and genotype will also affect the chemical constituents in the root exudate [47,48]. As mentioned above, these exudates are a blend of molecules, which are influenced by plant size, genotype, photosynthetic activity, and soil conditions. These phytochemicals influence the diversity and the composition of the microorganism around the root [49], where the amalgamated exudate modifies bacterial assemblage in the rhizosphere. When the influence of species was tested in the angiosperms, variation was observed in the *Pseudomonas* species occurring in the soil. This variation in soil species was also influenced by the spatiotemporal and physiochemical organization of the rhizosphere [25,50].

In addition to the microbes found surrounding the root system, there are also the endophytic microbes in plants. The root source components play deeply into the colonization of endophytes in the plants. Some of these endophytes have important symbiotic uses in agriculture. One example is the *Piriformospora indica*, which causes elevated phosphorous [P] uptake and protects against various stressors in plants [51]. Further, Gill et al. [51] reported that cyclophilin A–like from *P. indica* was overexpressed in protecting against salt stress in tobacco plants. When working in concert, *Azotobacter chroococcum* and *P. indica* help with nutrient acquisition and synergy in action [52]. Some of these endophytic organisms were also responsible in chemotaxis activities. In tomatoes, the non-pathogenic *Fusarium oxysporum* reduced the occurrence of nematodes [53]. When biochar was used in tomato plants, it absorbed the exudates and created a strong chemotactic signal towards *Ralstonia solanacearum*, suppressing its swarming ability [54]. Collectively, the mechanisms, functions, and communication signals in root–microbe interactions were reviewed in other publications, detailing the intricacies of these interactions [25,46].

#### 2.2.1. Root-Root Interaction

Plant species and genotypes have strong specificity of exudates that are able to influence the neighboring plants. Little is known on how these signals are transmitted and received by both root and microorganism. These root exudates have been implicated in several processes, including influencing nutrient availability [49] and mediating nutrient competition. Plant exudates have been reported to increase mineralization. The presence of certain acids in the soil (phosphatases, carboxylase) improved ion availability to the plant [55], and indirectly influenced N_2_-cycling. The absence of these acids in exudates will influence N availability in soils [56] explaining the difference in nutrient acquisition between plants. In addition, plants are not only influenced by its own exudates but are influenced by their neighboring plants. For instance, in intercropping where leguminous and non-leguminous plants are grown together, the release of carboxylates by legumes resulted in enhanced P nutrition and growth to neighboring plants [57]. In phosphorous deprived soils, Zemunik et al. [58] reported that the phosphorous levels were replenished through the influx of carboxylate exudates by arbuscular mycorrhizal fungi (AMF) and plant roots. In plants such as cucumber, citric acids from the roots attracted *Bacillus amyloliquefaciens*, and fumaric acid from banana roots attracted *Bacillus subtilis* resulting in biofilm production [59].

The root–root interaction is not limited to the regulation of nutrient acquisitions. These interactions also influence the root growth of neighboring plants through allelopathy, where released phytotoxins are able to reduce the growth or survival of neighbors and, therefore, reduce competition of resources. A classic example of toxins is catechin, which inhibits germination, root growth, and development [60,61]. Volatile organic compounds (VOCs) also function as allelochemicals to regulate rhizosphere signaling by mycorrhiza networks [62,63]. Allelopathic plants are mostly resistant to their own phytotoxins and, therefore, act specifically on other plant species at different levels of effectiveness. However, there are certain non-allelopathic neighbors that can be resistant [64]. The exudates around the roots are controlled by the rhizobiome, which affects the quantity, composition, and possibility of feedback regulation between the plant and microbiome. From the above observation, it would appear that the root–root interaction is one that is competitive and not niche [65]. Competition between neighboring plants is seen in species that have root systems spread across a wider region horizontally than those that have vertical and deep root systems. Furthermore, when legumes, non-legumes, and interspecies studies were conducted to evaluate root–root interactions, these plant exudates influenced each other and the microbial communities within their vicinity [66,67].

The root–root interactions may show the presence of major bacterial communities and AMF in the soil [36]. The bacteria may largely be responsible for hormone induced growth and antibiotics based on inhibition of negative organisms. The AMF, on the other hand, is an important component that plays an important role in water and nutrient absorption. This organism has the ability to maintain some level of nutrient absorption despite competition in soil; thus, it maintains the plant community composition [68,69]. In addition to the positive interactions found in the soil, the ecosystem is home to soilborne pathogens. These pathogens affect the plant soil feedback by affecting the plant growth, nutrient accumulation, and other processes, resulting in a prolonged negative impact on the soil microbial population due to the presence of the pathogen and the application of fungicide [70,71]. This will cause a shift on the hierarchies of different soil microbial communities, depending on the changes in the soil from environment, human activity, and disease. While it is still unclear on how pathogens affect nutrient uptake, it is possible that it negatively affects plants through root growth and resource uptake per unit root [72].

#### 2.2.2. Root–Microbe Interactions

The root–microbe interactions can be addressed as symbiotic and parasitic interactions. In this section, we explore the beneficial interactions established between the root–microbe, such as: [1] rhizobacteria–legume; [2] actinobacteria–root; [3] mycorrhizal–root, and [4] other root–microbe interactions; of these, the most widely studied relationship is between legumes and rhizobacteria. These organisms produce Nod factors, perceived by the plant receptors as inducing activation of the pathway, resulting in nodule formation from the differentiation of pericycle and cortical cells [25,73]. The bacterium then uses this organ as a processing site of atmospheric nitrogen into ammonia, which is then used in protein synthesis. This process is regulated by feedback inhibition to reserve energy and inhibit N_2_-fixation when the supply of nitrogen is sufficient [74].

As Rhizobium interactions are not established with every plant, certain model organisms have been used in understanding the changes that occur within the rhizobium and nodule. Larrainzar and Wienkoop [75], Lorite et al. [76], and Wan et al. [77] conducted a proteome analysis on the genome sequence of *Sinorhizobium meliloti,* a symbiont of *M. truncatula, Mesorhizobium loti*, the symbiont of *L. japonicus,* and *Bradyrhizobium japonicum*, the symbiont of soybean, and compared it with the free-living organisms. These analyses enabled them to answer some very important questions on the recognition of the host by the rhizobacteria, nutrient exchange, and the control of nodulation. It is believed that the flavonoids released by the legumes into the soil are able to activate the NodD protein, which in turn sets into motion a series of nodulation genes encoded by Sym plasmids. From the studies conducted by the above researchers and others, it is concluded that flavonoids are responsible for the varied responses of several bacterial genes.

The rhizobacteria provides signaling chemicals that act on their host. A study on *Medicago truncatula* disclosed that there was change in protein content within the nodule during the formation of leghemoglobin and enolase isoforms [31]. In other legumes, *R. leguminosarum* induced ethylene responsive proteins. Ethylene is a major regulator of plant defense response. In the ethylene-insensitive mutant of *M. truncatula* (*skl*), hypernodulation was observed in the roots, likely due to a compromised immune system. It was reported that the *skl* mutant had a defective ethylene pathway. Therefore, we hypothesize that ethylene is responsible for the symbioses and nodulation of the host. Further, when the root system in soybean was examined post inoculation with *B. japonicum*, there were rhizobial proteins that were necessary for the induction of the Nod factors detected in the roots [77]. Elevated levels of calcium-dependent protein kinase (CDPK) were observed and were expected to trigger the activation of symbioses [78]. The presence of peroxidases, lipoxygenases, phospholipases, and lectins indicate a possible role for lectins in attachment, and lipids in the early infection process of rhizobacteria in the root system [79]. While the success of the nodulation process is reliant on the lack of defense response against the rhizobacteria, a proteomic study of the *M. truncatula* root colonized by *S. meliloti* identified pathogenesis related protein (PR10) isoforms [80]. These proteins were implicated in phytohormone interactions, and ligand binding, influencing specifically auxin and cytokinin activity in plant meristem [79,81].

Nutrient exchange is another factor that influences the rhizobacteria-legume relationship. Rhizobacteria are present as bacteroids in the plant symbiosomes where all nutrient exchange is controlled by the composition of bacteroids and peribacteroid membranes. When the proteins in these membranes were analyzed, heat shock proteins, proteases, nodulins, transporters, receptor kinases, plant defense, and signaling proteins were identified, indicating that nodulation is an ongoing, complex process, where the plant’s defense mechanism is continuously regulated to allow for the nodulation in the roots [82]. The nodules contain enzymes required for C utilization, N_2_-fixation, heme synthesis, transporters, and stress related proteins. The differences shown by the ABC transporter in free-living and nodule inhabiting bacteria imply that they are responsible for specialized functions in nutrient transfer and nodulation [83]. The proteome studies are indicative that bacteroids enhanced nitrogen and carbon metabolism while suppressing fatty acid and nucleic acid metabolism [84]. However, transcriptome studies observed expression of high levels of aquaporins, ATPases, metal binding proteins, nutrient transporters (carbon, nitrogen, potassium, and sulfate), osmoregulators, and regulatory proteins in the nodules [85,86]. All of these components are useful in maintaining homeostasis within the nodule, to facilitate the transmembrane transport of nutrients and proteins.

The proteomes of nodules have also been studied under stress circumstances. Drought is a primary stressor that prevents nodules from fixing nitrogen. The metabolic enzymes, such as sucrose synthase, amino acid synthesis enzymes, and leghemoglobin, which regulates oxygen levels within the nodule, were reduced in drought-stressed *M. truncatula* nodules [87]. Drought results in an increase in protein accumulation in the bacteroid fractions, including enhanced protein synthesis components, in contrast to the decreased protein accumulation in the host [88]. As nodulation is a costly process for the plant, it is presumed that this process is auto-regulated through signaling mechanisms. A suggested mode of control involves auxin transport from the shoot to the root to regulate nodule numbers as seen from the differential expressions of auxin inducible proteins in mutant and wild type *M. truncatula* [89,90]. The second possibility is the regulation of the plant defense mechanisms that may arrest or inhibit nodulation [91].

Actinomycetes are also able to form symbiotic relationships with host plants. Some symbiotic relationships of this group of bacteria have been reported in Angiosperms, such as Alnus, Casuarina, and Datisca genera [92]. A study of proteomes in *Frankia alni* and *Alnus* sp. identified secreted proteins, which were generally hydrolytic enzymes believed to play a role in the formation of this symbiotic relationship [92,93]. Another group of organisms involved in the symbiotic relationship with plants is the mycorrhizal fungi. These fungi invade the root systems and establish the arbuscular in the cortical cells or extracellular hyphal structures (ectomycorrhiza or EM). The AMF are the most dominant of these fungal interactions [93,94]. Like in the rhizobacteria, AMF remains separated in the plant by a membrane, which does not hamper the nutrient exchange between the host and fungus. AMF provides the phosphorus to the plant in exchange for carbon and lipids [95]. The carbon supply to the symbiont is feedback-regulated to limit excessive loss of nutrients from the host [96]. A proteome analysis on the root of *M. truncatula* colonized by *Glomus mosseae* exhibited redox, stress, respiration, and cell wall modifications, all necessary changes to facilitate the colonization of the host root system by *Glomus mosseae.*

A differential expression of proteins was observed in wild type and mutant [*dmi3*] *M. truncatula* proteins inoculated with *G. intraradices* [97]. Proteins, such as lipoxygenases, thioredoxins, and ATPases, were identified through proteomic and transcriptome analyses. Further, studies on these proteins showed the importance of transporters (nutrient and water) [98], and metabolism (amino acids, fatty acids, and carotenoids) in AMF infected roots [96]. When the *G. intraradices* infected wild type, *dim3,* and *sunn* mutants of *M. truncatula* were analyzed, proteins that were specifically induced or reduced were chalcone reductase, a 2,4-D-inducible glutathione transferase, a glutathione-dependent dehydroascorbate reductase and a cyclophilin [97]. These proteins postulate the importance of this symbiotic relationship in redox and defense mechanism facilitation for healthy plant growth and stress management. With the presence of annexins, alcohol dehydrogenases, and profucosidases—there is the possibility of the mycorrhizal infection playing a role in detoxification, in addition to stress response [99].

One area of symbiotic relationship that is extensively studied is the relationship between free-living organisms, such as *Trichoderma* and its positive impact on plant host protection from disease, induction of immune response, and improved plant growth. *Trichoderma* has become an important biological control agent as this genus has the ability to parasitize other fungi through diverse mechanisms. *T. harzianum*, an extensively studied species, produces proteases that are able to degrade fungal cell walls. *T. asperellum* was known to induce the production of proteins related to disease and the defense response pathway [5,43,63,100]. Additionally, these organisms resulted in an increase in levels of isoprenoid, ethylene biosynthesis, energy metabolism, and protein folding. *T. atroviride, T. harzianum*, and *T. asperellum* were reported to have elevated levels of disease resistance proteins, such as chitinases and cyclophilins, which provides heightened resistance in the plants [101].

#### 2.2.3. Microbe–Microbe

Communication between microbe–microbe can be addressed as (1) between pathogenic microbes; (2) pathogens and endophytes; (3) succession by microbes; and (4) lifestyle changes in the environment. Pathogens are able to affect microbial community on plant surfaces and soil. In maize exhibiting SLB infection, it was observed that the resident microbial community was reduced in richness [102]. When infected by a pathogen, the host is open to infection by others, even non-pathogenic microbes due to increased susceptibility. When infected with white rust, *Brassicaceae* were more susceptible to mildew pathogens and, hence, easily succumbed to white rust [103,104]. In *A. thaliana*, *Albugo laibachii*, was observed to have increased susceptibility to non-host pathogen *Phytophthora infestans* [105]. Bacterial populations utilized quorum sensing (QS) and biofilm formation as a means to establish beneficial plant-microbe interactions [106,107]. QS mediated by QS signals between pathogenic organisms were implicated in increasing the pathogenicity and virulence of these microbes on the host [108]. One such example is when QS resulted in *Phytophthora nicotianae* zoospore aggregation, which resulted in heightened pathogenicity [64]. However, while interaction between the species of microbes in the infection process is evident, each microbial cell is responsible for the successful colonization and disease progression in a host [109]. Many genes have been identified in bacteria, responsible for the formation of biofilm, colonization of the roots, and improved growth [110]. The QS signals produced by bacteria are able to effect plant transcriptome and proteome [79] by adhering to the environment and plant surfaces and, thus, impacting the processes within the plant [111].

Busby et al. [112] observed that foliar pathogens might be inhibited by endophytes through hyperparasitism, competition, and/or antibiosis. These endophytes produce a list of chemicals that are toxic to microbes and can prevent pathogen infiltration [113]. Some of these interactions between endophytes and pathogens are direct. Jakuschkin et al. [114] reported in his study that fungal endophytes acted antagonistically against powdery mildew of *Erysiphe* sp. The presence of chemical constituents, such as polyketide synthase, are natural antibiotics in endophytes that enable these organisms to act as biocontrol agents against pathogens [115]. *T. atroviride*, *Ulocladium atrum*, *Stachybotrys* sp., and *Truncatella angustata* were shown to generate quantitative disease resistance in *P. trichocarpa* against Melampsora rust pathogen by Raghavendra and Newcombe [116]. The afore-mentioned fungi, on the other hand, were found to be relatively uncommon in wild *P. trichocarpa* [112], suggesting that disease-modifying effects of foliar fungus differs between wild and experimental settings. Endophytes also employ QS to inhibit harmful bacteria through the expression of QS inhibitors (QSIs) or quorum-quenching (QQ) enzymes to prevent signaling molecules from working. The plant pathogens *Erwinia carotovora*, *Bacillus thuringiensis*, and *Enterobacter asburiae* have all been inhibited by the AHL lactonase enzyme (a powerful QQ) found in endophytic bacteria [117,118]. Enzymes produced by bacteria protect plants against environmental and biotic stressors. In drought, trehalose helps stabilize the membranes and enzymes. Surplus supply of trehalose by bacteria not only helps alleviate environmental stresses but also helps with eliciting disease response and induction of systemic resistance (ISR) [119]. Further research is needed to decipher how these interactive chemicals impact the plant microbiome structure and function and influence the plant health [120].

Microbes that colonize a host will always compete for nutrient, space, and survival. It was observed that the order of infiltration decides the resistance of the host against the infection. For instance, if an endophyte is present within a host before the arrival of a pathogen, the resistance will be stronger compared to when the pathogen and endophyte infiltrate the plant together or if the pathogen arrives slightly before the endophyte [121]. The biotrophic pathogen *Ustilago maydis* was inhibited by co-inoculation with *Fusarium verticillioides*. The endophyte did not protect when applied prior to the infection, indicating that the endophyte inhibited *U. maydis* by direct interaction. *U. maydis* did not affect the endophyte community, and it did not relate to the differences in the levels of resistance in the maize lines [122].

**Figure 1:**
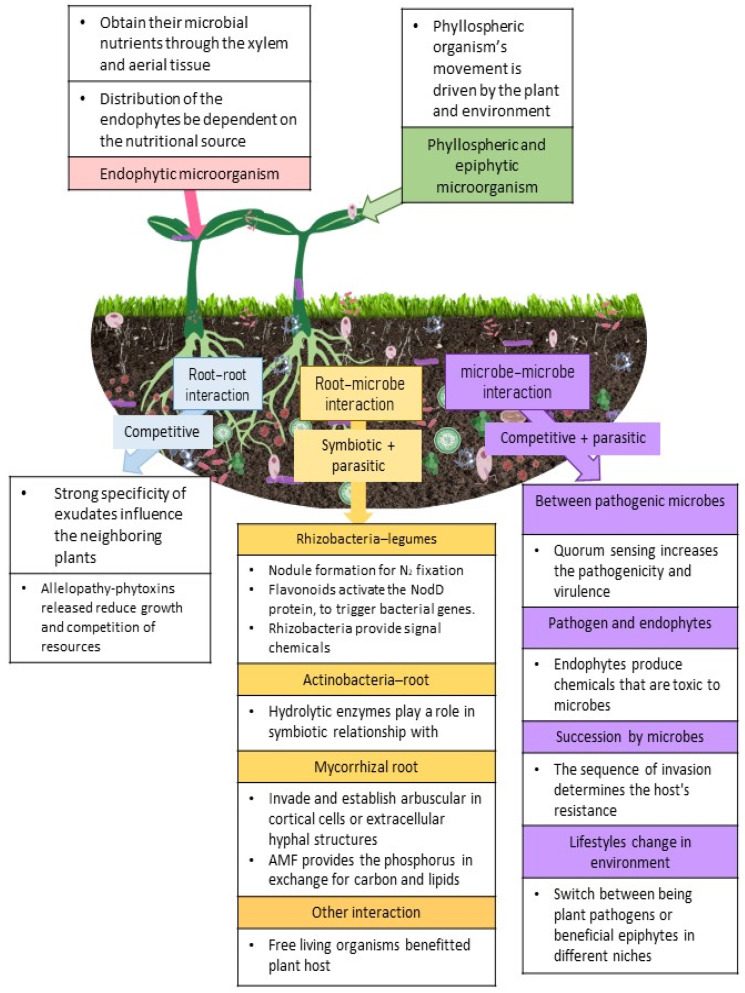
Shows the plant-microbe interactions aboveground and belowground observed in a plant’s natural environment. The pink box covers endophytic microorganisms, whereas the green box describes phyllosphere and epiphytic microorganisms. The purple column provides the explanation for microbe–microbe interaction; the orange column shows the explanation for root–microbe contact, and the blue column for root–root interaction. 3. Microbes and plant immunity.

*U. maydis* is an interesting organism that has the ability to exhibit different lifestyles in different niche environments. There are other organisms that display such characteristics, for instance *Moesziomyces* sp. and *Ustilaginales* act as biocontrol agents in certain niches [108] through the secretion of hydrolase that antagonizes *A. laibachii* [123]. However, it was reported that some of these *Ustilaginales* could switch between being plant pathogens or beneficial epiphytes in different niches. This is also observed in instances where certain *Fusarium oxysporum* can act as antagonists to other *F. oxysporum* strains [124]; this was linked to the plethora of effector molecules produced. However, effector molecules have not been identified from endophytes and cannot be linked to any host specificity [125]. Therefore, this suggests that the anamorphs of *Ustilaginales* may produce filamentous structures [126], but there is no clear indication as to what the different adaptations in these organisms are that make them switch between pathogenic and epiphytic lifestyles. Figure 1 provides the plant-microbe interactions that are generally observed aboveground and belowground.

Plants are an important source of nutrient for microorganisms. When microbes form non-beneficial interactions with the host, the immune system of plants would be triggered, either strongly or weakly, depending on the host and the pathogen. Unlike animals, plants have a defense mechanism, with structural, chemical, and protein-based components, to defend against attacks. A good understanding of the plant immune systems will allow us to develop better disease resistant varieties. Unlike our mobile and adaptive immune systems, plants depend on innate immunity, efficient signaling pathways, and beneficial microbes [127]. The initial step in triggering a defense mechanism lies in the invasion of the host cells. Pathogens, such as bacteria, infiltrate the host through various mechanisms, e.g., trichomes, lenticels, stomata, and other openings. However, in fungi, the infiltration process depends on the formation of the penetration pegs, while viruses are opportunistic pathogens that enter through injuries or locations of infection to cause disease in the plants [128,129,130].

When the primary defense is breached, the microbe associated molecular pattern (MAMPs/PAMPs) activates both the MAMP-triggered immunity/PAMP-triggered immunity (MTI/PTI) and the effector-triggered immunity (ETI). MTI/PTI is the horizontal immunity, while ETI is the vertical immunity. Some pathogens may trigger the ETI without the PTI through the interaction of effector molecules and the nucleotide-binding site-leucine-rich repeat (NB-LRR) found in the R genes, resulting in hypersensitive cell death [HR] [130]. While PTI and ETI share some common chemical components, they are viewed as separate evolutionary pathways [131] that are responsible for the plant’s immunity. A single NB-LRR receptor (directly or indirectly) provides immunity against pathogens once activated by pathogen effector molecules. The PTI involves protein recognition receptors (PRRs) that are present on the cell surface that act as binding sites for PAMPs/MAMPs. Consequently, the bound complex elicits a signaling cascade that is responsible for inhibiting the growth of the pathogens/microbes [130,132]. While plants have the PTI and ETI, microbes have evolved mechanisms that are able to overcome the PTI, by releasing effector molecules into the plant, triggering plant susceptibility.

Previously, it was assumed that the presence of the *R* gene was necessary for the perception of the pathogen. This was alluded to as the guard model. Recent research has shown that the indirect recognition of the effectors is inconsistent with the guard model. Presently, it seems that multiple recognition sites are available for different microbe effectors. It is now well-established that multiple targets in hosts are present for different pathogen effectors and the classical Guard Model does not explain this when lacking the R protein [133]. What is observed above involves evolution and, therefore, would be better explained by a decoy model [134]. The decoy is explained as a concept where the effector target is the decoy that acts on pathogen perception, even when the R protein is absent [133,135]. At the point of infection, systemic acquired resistance (SAR) is activated to prevent further proliferation of the pathogen to neighboring cells through the activation of the defense pathway, which results in the activation and expression of pathogenesis-related (PR) proteins [136,137].

Through the advent of the genomic tools, a better understanding of the interactions between plant and pathogens is obtained. Transcriptomics have enabled us to identify genes that are enhanced or inhibited in the plant-microbe interaction, providing a clearer picture of what may be happening in the regulation at the molecular level [25,138,139,140]. These studies also implied important roles for microRNAs in plant response against the pathogens, the plants innate immunity, as well as the triggered defenses in plants [141,142,143]. Beyond the effectors, receptors, and models described, it was postulated that the immune system in plants is moderated by systemic and local elicitation of phytohormones. These hormones are involved in the activation of induced systemic resistance (ISR) and SAR. For the above responses to take effect, there has to be interactions between the plant and the microbe [37,137,144,145,146].

SAR is split into several steps, where the most significant stage of SAR response is signal production and amplification at the site of infection and signal transduction to distal organs [147]. Numerous mobile chemicals were discovered as potential SAR signals or significant contributors in the mobility of long-distance SAR signals. Among them are methyl salicylate (MeSA) [148], glycerol-3-phosphate dependent factor (G3P) [149], azelaic acid (AzA) [150], dehydroabietinal (DA) [151], the lipid transfer protein known as defective induced resistance 1 (DIR1) [152], and pipecolic acid (PIP), a lysine catabolite amino acid [153,154]. Following signal detection, the emergence of SAR (defense priming) in the distal organ is linked with extensive metabolic and transcriptional remodeling [149,155].

In Arabidopsis, the important molecules that must be present in the distal pathogen free leaves are the buildup of PIP and SA, followed by the expression of flavin-dependent-monoxygenase1 (*FMO1*), enhanced disease susceptibility (*EDS1*), flowering locus D1 (*FLD*), isochorismate synthase 1 (*ICS1*), phytoalexin deficient 4 (*PAD4*), AGD2-like defense response protein 1 (*ALD1*), and SNF1-related protein kinases 2.8 (*SnRK2.8*) genes. The majority of these components are parts of the SA-amplification chain [156]. The activation of a transcription factor, a non-expressor of PR genes 1 (*NPR1*) by SA is also required for defense priming [154,157,158]. It must be emphasized that defense priming and signal amplification are interdependent with systemic PIP formation and PIP facilitated SA-independent and SA-dependent priming of plant defenses in an FM01-dependent manner [154,155].

**Figure 2:**
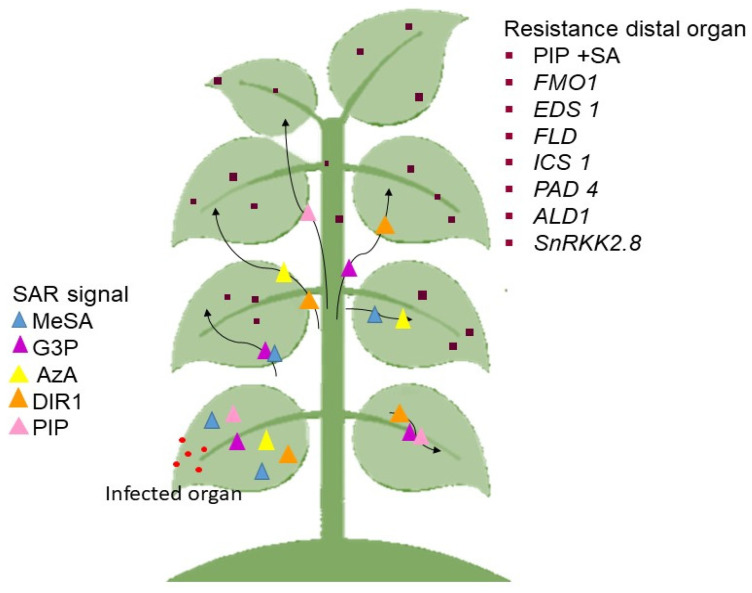
The process of the SAR mechanism. The generation of signals in the diseased organ. The signal is sent to other parts of the plant that are not affected. After transcriptional and metabolic remodeling, the essential molecule is present in the healing organ as immunity. The black arrow represents the movement of the SAR signal to the distal organ.

Previous findings also showed that the SAR-inducing action in cucumber and Arabidopsis phloem sap caused by various phytopathogens proved efficacious in other plants [149,151], suggesting that the mobility of SAR signal(s) are not unique to plants or pathogens [156]. The SAR signaling transmission is as seen in Figure 2.

While SAR happens at the site of pathogen infiltration, ISR happens from its site of trigger in the rhizosphere. The association between the plant and microbe in the soil can be used to improve plant defense and crop productivity. When disease is present in the soil, microbes with the ability to inhibit the activity of these pathogens may be introduced to manipulate the environment into one community that ensures the health of the soil. This may be achieved with a rich inoculum and the management of environments [136,159,160,161,162]. Finding the right balance in plant-microbe and microbe–microbe interaction is important in establishing chemical communication in the rhizosphere. This association plays an important role in engaging the signaling cascade that prompts the resistance or defense in the plant against pathogens and facilitates activities that improve yield and growth of the crop [131,163,164].

The microbial assemblage in the soil secretes molecules that are able to induce gene expression in the plant species. Some of these signals (VOCs, for example: alcohols, alkanes, ketones, terpenoids, etc.) operate as communicators within the microbial communities in the rhizosphere [25,131,165]. These compounds promote several functions, such as disease inhibition, nutrient acquisition, improved growth and development, mineralization, and other processes. These compounds are also responsible for triggering alterations in the plant’s transcriptome. While phytohormones, such as auxins, abscisic acid (ABA), cytokinins, gibberellins, jasmonic acid (JA), and salicylic acid (SA) are at play in plants; the same hormones are also secreted by beneficial microbes [145,166,167,168]. The beneficial chemicals exuded by microbes that activate plant defense mechanisms are listed in Table 1. In addition to the induction of ISR by the plant-microbe interaction in the rhizosphere, immunity or plant defense may also be elicited through a phenomenon called *trans*-generational. This form of immune memory is transferred to the following generations in the plant in response to pathogens [131,169]. For instance, when an avirulent *Pseudomonas syringae* was applied on *Arabidopsis,* it resulted in the next generation of plants, showing increased levels of salicylic acid [SA], which resulted in heightened disease resistance [131,162,169].

Despite many remarkable discoveries in the field of plant immunity, many mysteries remain unresolved, such as the identification of avirulence (*Avr*) genes in plant–pathogen interactions, plant root immune mechanisms, molecular mechanisms of pathogen colonization in plants, regulation of cellular activity and gene expression, and signaling mechanisms involved in plant immunity. As a result, progress in post-genomic era technologies will open the door for a deeper understanding of plant–pathogen interactions and plant immunity.

## 3. Functions of Rhizosphere Consortia

As mentioned in the previous section, soil microbes are involved in three main processes—growth and development, nutrient acquisition, and stress management against biotic and abiotic stressors. These processes are managed through an interplay or chemical signaling that facilitate these functions.

### 3.1. Hormones and Their Promotion of Growth and Development

Firstly, organisms, known as plant growth promoters, achieve their purpose in plant growth and development through an array of phytohormones secreted into the soil. The main players, such as auxin, cytokinin, ethylene, and gibberellin influence plant growth, and are extensively studied in cereal root systems [8,136,203]. *Pseudomonas*, *Burkholderia*, and *Pantoea* are involved in biological processes, such as P solubilization, N_2_-fixation, auxin, and ACC deaminase production. Beneficial and non-beneficial organisms produce auxin. Auxin is responsible for root growth and formation, elongation of nodular cells, and response against stressors [204,205,206]. In pathogens, auxin is linked to its virulence. One good example of auxin’s role in virulence has been studied in the *Agrobacterium tumefaciens* where the expression of tumors in plants depends on the secretion of IAA [207,208]. As tryptophan is required for auxin production, aminocyclopropane-1-carboxylic acid [ACC] is needed for the production of ethylene and microbial growth. ACC deaminase-producing PGPRs help in the utilization of ACC to equilibrate the levels of ACC inside and outside the plant [209,210].

Ethylene is involved in the regulation of growth, elicitation of defense, and the management of plant stressors [211]. The elicitation of defense response by singular or consortia is dependent on ethylene. The role of ethylene in affecting the community structure was determined using ethylene mutants. These studies showed that the mutants affected the bacterial community structure, but these studies were not able to correlate the abundance of species to ethylene due to variable ethylene levels, and the cross-talk with other hormones [211,212,213]. Moreover, the original colonizers will have the ability to influence the microbial population. Hence, if the microbial populations are linked to ethylene regulation, they are likely to shape the microbial structure in the soil and control the regulation of stress in the plants [36,136].

Jasmonic acid (JA) and its methyl ester (MeJA) have been associated with defense and wound response in plants [214,215]. Recent studies have also alluded to JA being involved in the recruitment of microbial communities around the roots [8,163]. JA regulates the components in the root exudates, such as benzoxazinoids, known to improve herbivore resistance. These compounds contribute to the allelopathic and chemotactic nature of root exudates. These exudates recruit miscellaneous microorganisms that cater to specific niches in the soil as well as the specific needs of the plant itself [164,216]. However, while JA is responsible for the recruitment of microorganisms, we are unable to correlate JA and the population structure due to too many variables in the environment.

Salicylic acid (SA) is another signal molecule that is involved in plant defense. However, unlike JA and ethylene, SA is directly related to SAR. Together with JA and ethylene, SA forms the core defense hormone in the plant. The role played by SA has been studied using *A. thaliana* mutants, where the knockout mutants showed lower levels of survival and less prolific colonization [136,217,218]. Further, the study by Lebeis et al. [219] observed that SA linked pathways were required for the colonization of endophytes and the shaping of soil microbial structure. However, phytohormones (ABA, cytokinin, ethylene, JA, SA IAA, brassinosteroid, and others) may show synergistic or antagonistic effects against plant related processes. For instance, ABA is a major player in moderating abiotic stresses. ABA negatively interacts with SA mediated defenses and works either positively or negatively with JA and ethylene related biotic responses, respectively [41,220,221]. Therefore, in either biotic or abiotic stressors, phytohormones play their specific roles in shaping the soil microbial structures [25].

### 3.2. Biological Processes in Nutrient Acquisition

Microorganisms are involved in nutrient cycling and acquisition from the soil. Therefore, organisms, such as plant growth promoting rhizobacteria (PGPRs), are studied extensively for use as biofertilizers [222]. The compounds exuded by these microbes and plants work together to facilitate processes, such as nodulation, quorum sensing, N_2_-fixation, mineralization, and others [223]. Some of these processes have already been discussed under plant–microbe interaction Nod factors bind lysin motif-containing receptor-like kinases (LysM RLKs) and initiate signalling cascades, resulting in nodulation by bacteria in exchange for photosynthetic carbon [25]. Further, bacteria that establish IAA secretion in and around the root area enable the development of root hairs [223,224].

There is evidence that the biochemical constituents of rhizobacteria are able to elicit defense as well as facilitate symbiotic relations. Iron, for instance, when secreted by certain *B. subtilis* strains, is able to activate the host defense mechanism [35,46,136]. Bacterial volatiles activate the Fe deficiency transcription factor that, in turn activates a series of enzyme that results in iron accumulation. Freitas et al. [225] observed that when G03 was used to treat cassava plants, iron content increased substantially in the leaves. Similarly, certain organisms, such as *Bacillus paramycoides* KVS27, and *Bacillus thuringiensis* KVS25, increased growth of *Brassica juncea* through P solubilization, N_2_ assimilation, IAA, siderophore, and HCN production. The observed activities were attributed to the synergism among these organisms that resulted in the secretion of multiple chemicals, collectively resulting in plant growth. Therefore, the effects that are seen on plant defenses may involve a consortium rather than singular microbes. The interaction between microbe–microbe, microbe–plant, and microbe–environment collectively influences growth and development of plants and the microbial community [226].

### 3.3. Microbial Defense Mechanisms

The manifestation of disease depends on various factors, such as host range, susceptibility of host, environment, pathogen population, agricultural practices, and various biotic stressors [227,228]. The resistance towards any pathogen produced by a host depends on the roles played by aboveground and belowground microbes that are able to modify the defense responses in plants [73]. Though the control of disease has been largely through chemicals, the effort to go green has directed research in the identification of biocontrols in disease suppression [35,71,229,230]. The use of beneficial microbial population is slowly gaining popularity worldwide, where enzymes, antibiotics, siderophores, volatile compounds, and inhibitory chemicals control the spread of disease [231,232,233]. These biocontrol agents have a myriad of activities that enable them to suppress the pathogens. Whether it is antagonistic, competitive, or triggering of the defenses—all work well in keeping disease in check. The antibiotics that are expressed by microbes promote growth and suppress pathogens. This is achieved through the activation of certain hormones, such as auxins, which enable changes to root architecture to improve nutrient absorption and improve growth [234]. Pseudomonads are widely known to produce DAPG, which induces ISR, while cyclic lipopeptides (cLPs-surfactin, fengycin, and iturin) from *Bacillus* spp. and *Pseudomonas* spp. produce surfactants that are able to inhibit pathogens [44,235,236]. In addition to the antimicrobials, and lipopeptides, QS enzymes play a role in suppression of disease, and induction of ISR [35,237]. Some of these organisms also play a role in regulating defense through the control of hormones in plants [238]. While certain taxa, such as *Actinobacteria, Serratia,* and *Enterobacter* are able to control several soil-borne diseases. These groups of organisms are able to induce action through ISR and SAR, protecting the plant systemically through the involvement of hormones, signal molecules, and the activation of pathways in the plant [239,240].

## 4. Challenges of Emerging Plant Pathogens and Their Impacts on Plant–Microbe Interaction

As addressed in sections above, plant–microbe interactions can be either positive or negative. However, an immediate challenge to agriculture is the new and emerging pathogens that continue to plague the industry. While plant defense mechanisms are in place to protect the plants from the exposure to pathogens, new and emerging pathogens may have evolved mechanisms that enable them to evade the host’s innate immune system [241]. Since it has been observed that pathogens co-evolve with their host, it is likely that, to avoid this “arms race”, the pathogens expand their host ranges. These organisms evolve their virulence or pathogenicity factors to enable them to elicit disease in the same susceptible host and new ones [242]. These new and rapidly evolving microbes pose a threat to the agricultural industry. A better understanding of the pathogen’s invasion and infiltration strategies will enable better control over disease through strategic heightening of defenses or breeding [127].

Several possibilities for the emergence of new harmful organisms (such as bacteria and fungus) are (i) the bacteria may be endemic in agricultural land, but a novel host has just been found, (ii) After becoming endemic, the microorganism turns pathogenic, owing to a rise to its pathogenicity or a loss in the host’s defenses, (iii) The microbe may have just been introduced into a new environment with unknown hosts, and the organism might be harmful to novel plants, and (iv) Insect vectors feed on a new host, containing harmful organisms, and spreading the organism to succeeding plants [243]. Diseases arise due to a variety of causes, including interactions between pathogenic organisms, plant–pathogen interactions, plant–insect–pathogen interactions, and unfavorable environmental circumstances. According to Deberdt et al. [244], climate change, can modify the character of microbes, transforming them into opportunistic diseases. It is well known that when plants are weakened or stressed by external conditions, microbes may easily colonize them, resulting in plant mortality. Certainly, various abiotic stress, such as drought, heat factors, and so forth affect the plant, inflicting great damage to the forest and agriculture [245].

Further, trade has become an agent of disease transmission globally. Though scrutiny of migration pathways and quarantines have been imposed, new and emerging diseases are constantly being reported [246]. The advent of omics tools has enabled us to obtain new information on emerging populations. There is also a flood of databases of phytopathogens and plant genomes that has made it possible for us to study the plant-microbe interaction more closely [247]. The current information derived from the sequence databases show that there is an accelerated genome adaptation in pathogens to their environment. This high rate of evolution has further compounded the problem of disease in host–pathogen interactions. Population genomics studies is a good way to study the adaptive evolution of plant pathogens and design better disease management strategies. In the following section, we will deal with the technologies used to study the microorganisms and the plant-microbe interactions [248,249].

## 5. Unraveling Plant–Microbe Interaction at the Molecular Level

The underlying theme in this review involves the three main interactions observed between plants and microbes. While symbiosis and mycorrhizae are two main facets of this interaction, the aspect of disease has garnered interest, especially with the losses incurred by current pathogens and the threat of emerging diseases. The exploitation of this interaction provides for the development of sustainable disease management strategies [35].

While our current method of addressing disease in plants is through resistance breeding via conventional or molecular techniques, the advent of new genome platforms has enabled us to acquire large amounts of big data on plants and pathogens through a series of sequencing and re-sequencing of these genomes. The method of identification, such as 16S rRNA sequencing, WGS, or classic culture techniques, can potentially have an impact on the reporting of the discovered microbiome. Delmont’s [250] 2009–2012 survey of Park Grass, for example, employed at least six distinct techniques of DNA extraction to produce an accurate representation of the soil microbiome. The genomic and post genomic era is upon us, and we are now faced with this large amount of data that needs to be deciphered and utilized in the development of disease resistance in plants, as well as in improving our understanding of ISR and SAR [249,251,252,253]. It is now possible to dissect and scrutinize the plant-microbe interaction at a molecular level through the utilization of platforms of genomics, proteomics, transcriptomics, and metabolomics. The genome data on microbe and plants, the various proteins that are secreted in the plant–microbe interaction, and the differentially expressed genes in the host and the metabolomes involved helps us understand these complex relationships [254,255]. While the genome information may be utilized to develop resistant plants through breeding or genetic engineering, the protein information may be utilized to identify key proteins in plant growth and development that controls various physiological and biochemical pathways [79]. The transcriptome data enables us to observe the variations in the expression of genes in response to the environment, growth, and development, while the metabolome data provides us with the metabolic changes incurred through the interaction between the plant and the microbe. Collectively the post-genomic era data have enlightened us in the area of gene discovery, beneficial microbes, and proteins that may be used in crop improvement, growth improvement, and heightened disease resistance [256]. Below, we will briefly go through the techniques that are useful in deciphering the biological functions and benefits of plant-microbe interaction.

### 5.1. Genome Sequencing

The various genome-sequencing platforms that have been developed over the years have made studying the interactions between plants and pathogens, at the molecular level, possible, and more informative. The availability of genome sequences of plants and microbes and the ability to conduct genome wide annotation of proteins and genes through bioinformatics platforms has further advanced the field of plant–microbe interactions [257]. The very first contribution to the bacterial genome was first obtained in 1995, which resulted in the ability of computational modeling in envisioning the entire operation of this organism from the sequence structure alone [258]. In 2000, the sequencing and annotation of *Arabidopsis* paved the way for better understanding of the sequence to operations through the use of genome scale modeling and annotations [258]. Through genome informatics, we are able to understand the microbe and plant systems better at the molecular level. Genome sequencing also allows us to connect the multifaceted signaling pathways that regulate the defense mechanism in the plant. However, despite the large amount of data available from the genomic- and post-genomic era, there are still gaps in the knowledge due to the high complexity of the interaction between plants and microbes, complicated further by internal and external regulatory factors [259].

Generally, the genomics and transcriptomics data allows us to draw information necessary for the metabolic network modeling of plants and pathosystems. By merging the metabolic pathways of the plant and pathogen, we are better able to study the positive and negative effects of these interactions [249,257]. The initial study of plant–microbe interactions and understanding of the relationship at the genome level was taken one gene at a time or one protein at a time. Over time, a more holistic approach was taken where the entire plant and pathogen genome was elucidated together. In the early 21st century, transcriptomic tools, such as the cDNA microarray and SuperSAGE, were used to profile gene expression and signaling in *Arabidopsis thaliana–P. syringae* and rice–*Magnaporthe oryzae* interactions [95,260,261]. As the sequencing platforms became more advanced, the RNAseq technology was developed, and the differential expression profiles of plant−pathogen interactions were elucidated. Combined with the transcriptomics data, the proteome data of plant–microbe interactions were also derived through 2D gels, MS/MS, GC/MS, LC/MS, and iTRAQ [262].

One of the important outcomes of the post-genomic era is the utilization of the sequencing data in annotations, making sense of how the organisms operated through metabolic modeling [258]. Through these modeling activities, we are able to investigate the capabilities and inefficiencies of an organism through studies from the genes, to proteome and transcriptome [258,259]. Through these network models, we are able to address all possible interactions between plants and pathogens. Genome-scale reconstruction models (GSRMs) were developed for many organisms and are useful in understanding the multi-cellular community interactions for phenotype−genotype gap bridging, and to investigate the functional evolution of metabolic and regulatory networks [263].

### 5.2. Amplicon Sequencing

High-throughput sequencing of marker gene amplicons is commonly used to clarify the composition, structure, and geographic dispersion of microbial populations in the environment, and remains a popular method in plant microbiome research [251]. Amplicon sequencing has the benefit of being very precise, identifying specific groups of microorganisms or functional genes [264]. Amplicon sequencing specificity enables it to accurately identify many rare species; yet, its sensitive characteristics makes it susceptible to contamination [265]. Hence, any analysis that depends significantly on amplicon sequencing must include both positive and negative controls [266]. This technique involves the sequencing of PCR products, obtained by using primers for the taxon-specific variable regions [267].

When studying bacterial populations, the 16S rRNA gene is the target used for amplification, sequencing, and identification of the targeted microbiome [268]. Several different primer sets were developed for the 16S rRNA genes of bacteria and the 18S rRNA genes and ITS segments that surround their regions of diversity. The universal primers used in amplicon sequencing amplify genes from various taxonomic groups with varying degrees of effectiveness [269,270,271]. Given their length, 16S genes with large introns may be overlooked by standard PCR design [272,273]. The quantity of rRNA gene clusters per genome has a direct influence on determining the total relative abundance of specific bacterial species [274]. The amplified product is then subjected to any one of the sequencing platforms that are available [267].

The sequence information obtained from the amplified products can be used in phylogenetic studies of the organisms within the sample. The phylogenetic relationship derived can be used in inferring taxonomic information. This taxonomic identification is largely dependent on how extensive the reference databases are. While 18S rRNA and ITS is available for the identification of fungi, the ITS is preferred as there are good reference databases and the sequences from ITS show a higher level of variance [275]. Detailed categorization of observed reads to the genus or species level is sometimes challenging because the amplicon sequence lacks the necessary sequence diversity to identify closely related genera or species with 18S rDNA primers, [276]. As a result, the ITS region was recommended over the 18S rRNA gene because of the greater sequence diversity seen in the ITS region and the availability of a much more curated and extensive reference database [275]. Nonetheless, unequal ITS fragment lengths may encourage PCR amplification of shorter ITS sequences as an alternative. This might result in a skewed estimation of the relative abundances of fungal species. However, to make sure that there are no biases of relative abundance of fungal taxa based on ITS sequence identification, non-ITS based targets may also be included to provide robustness to the data derived [277]. Following amplicon sequencing, the microbiome is analyzed through clustering of OTUs based on the defined sequence similarity thresholds. Sequences with similarity are assigned to the same taxa by OTU. These microbes are assumed to share origins.

Although amplicon sequencing may be used to infer community function, it is not an ideal method unless particular functional genes are utilized, where the function and phylogeny are congruent [278]. The following issues are linked with the amplicon sequencing approach: (i) During DNA amplification, sequencing mistakes and chimaeras can occur [279]. (ii) It is possible that primer coverage will not cover the necessary microbial diverse populations [269]. (iii) The relative abundance of operational taxonomic units (OTUs) may be skewed due to differences in amplification efficiency across the target genes [280,281]. (iv) Variability in gene copy numbers may have an impact on conclusions based on the relative abundance of the OTUs [282].

### 5.3. Metagenomics

Metagenomic analysis provides a variety of approaches that are based on biomolecules, such as lipids, DNA, RNA, and proteins [283] that researchers may use to uncover plant microbiome activity and diversity to identify microbial participants in soil. The shotgun genome sequencing method of metagenomics, as opposed to the amplification of targets in the amplicon sequencing, provides more information. This method provides sequences from bacteria, viruses, archaea, phages, and fungi. However, in comparison to the 16S rRNA method, this technique will require higher information depth to distinguish the uncommon/rare members of the microbiome, and quality control to trim and filter the reads using bioinformatic tools [284]. The online-based tools are easily used for any sequence information and can be easily utilized to map the reads obtained against any reference databases. These mapped reads are then functionally annotated using various online resources [285].

The shotgun metagenome sequencing makes it possible to study greater structure of microbial communities while also providing an unbiased perspective of the phylogenetic and functional makeup of environmental microbial populations [286]. Through metagenomics, the level of identification can go right down to the strain level, which is at higher efficiency compared to amplicons, which are more likely to provide characterization to the taxonomic levels of the amplicons [287]. However, while the identification is more precise, this method would require additional bioinformatic tools to reconstruct the genome based on the short reads obtained, or the utilization of higher resolution sequencing platforms. The metagenomic method is a useful tool to find and characterize microbes at the strain level, where the algorithm used will enable the system to overcome the intergenomic repetitive elements and detect small differences in the genetics of the organisms [288,289,290]. Further, the gene sequences in metagenomics may be functionally annotated to provide a clearer picture of the microbial characterization compared to the amplicon survey. The functional annotation will include gene prediction and annotation, where, firstly, the protein coding sequences are identified, followed by matching this predicted protein to a protein function [291,292]. However, the identification of genes from the metagenome analyses does not ensure that all genes identified are expressed. While both amplicon sequencing and metagenome use the sequencing platforms, these methods have their limitations. This is precisely why sequencing platforms and bioinformatic tools are constantly updated and upgraded to improve the quality of reads and informatics obtained [293]. Therefore, to gain a better understanding of the total microbial diversity, studies may employ one or a combination of methods to acquire as much information as possible while adhering to their sample size.

### 5.4. Soil Proteomic

Proteomics is used in the study of the function and control of biological systems based on the prediction of protein profiles. Considering that soils have the capability to restore extracellular proteins through a variety of ways, the effectiveness of protein retrieval from diverse sources must be evaluated as part of the progress of soil proteomics [294]. Although metagenomics enables the identification of microbes in the rhizosphere, metaproteomics enables the investigation of rhizosphere biological activities [295]. It is feasible to relate ecological function to microbial community composition when these two techniques are used for the same problem [295]. Previous studies that used this technique attempted to comprehend the truffle brûlé mechanism in its specific niche, where other symbiotic fungi were driven away by this fungus once it formed symbiotic relations with the plant [296]. Recent research conducted on soil microbes have combined various omics methods (culturomics, metaproteomics, and 16S rRNA sequencing) to identify microbial communities and elucidate microbial population roles in the glacial ecosystem [297].

The idea behind mass soil protein analyses is that having a full proteomic profile of a microbial community would make it easier to find distinctive polypeptides whose syntheses are influenced by certain ecological factors [298]. Due to a huge number of unique proteins synthesized by various species, molecular characterization of soil proteins, for revealing species composition and metabolic activity, has been challenging. Amidst this drawback, advances in immunological methods, as well as a spike in the range of accessible enzyme analyses, have been utilized to complement precise molecular resolutions in situations where a specific polypeptide was of interest [299]. Understanding ecological activities by measuring molecular diversity in soil settings requires the understanding of protein structural complexities in comparison to other identifiable compounds, such as fatty acids and nucleic acids. One- or two-dimensional polyacrylamide gel electrophoresis (PAGE) can be used to create comparison protein profiles, relying on electric charge and physical size attributes [298]. Concerns of evolutionary variety within particular groups of species inhabiting comparable ecological niches may also be addressed using amino acid sequence analysis. Despite the fact that the metaproteomics approach has been around for more than a decade, it is still constrained by computational and technical support [295]. First, contamination by humic acid and other pollutants that impedes protein extraction makes it extremely reliant on soil type. Secondly, various extraction techniques might have an impact on the detected metaproteomics [300]. This limitation can be circumvented by utilizing several extraction techniques simultaneously and pooling all extracted proteins prior to further analysis [295]. Thirdly, protein identification is hampered by the lack of a comprehensive protein database [301,302]. Building in-house libraries largely depends on the metagenomics data acquired from comparable settings in previous studies [303]. The current availability of low-cost high-throughput sequencing has undoubtedly aided the integration of metagenomics with metaproteomics. However, by using next generation sequencing (NGS), it is feasible to get more reads in less time, allowing the species to be identified, and at the same time establishing an optimized databases for protein identification [295].

Overall, metaproteomics is a strong tool used for studying biological functions of a microbial community, and this information is used to correlate functional and taxonomic soil makeup in the ecosystem [304,305]. Furthermore, soil protein analysis might provide relevant data on the biogeochemical capacity of the soil and pollutant decomposition, as well as operate as a predictor of soil health and restoration [306]. This might help us comprehend organic contaminants and organic compound degradation, nutrient cycles, and plant–plant and plant–microbial interaction at the molecular level.

## 6. Microbes in Sustainable Agriculture

Microorganisms have a huge impact on the physical, chemical, and biological processes in the soil that are directly and indirectly important for plant and animal growth and development. While extensive studies have been carried out on a global scale to identify suitable microbes for use in the agricultural industry, more can be done in the continuous isolation and characterization of future biocontrol and growth promoting organisms that are suitable for specific applications. In this section, we will look at how bacteria can be used in agriculture.

Nutrient cycling: microbes recycle several nutrients, such as carbon, nitrogen, phosphorus, potassium, zinc, calcium, manganese, and silicon on a constant basis. Nutrient recycling is critical, not only for plants, but also for all forms of life, as it provides essential components for the synthesis of amino acids, proteins, DNA, and RNA required by all living organisms. The contribution of microbes in this regard is largely undervalued. Identifying and maintaining the density and community of essential microorganisms in each cycle will be of utmost importance. Further, to boost the organism’s cycle abilities, key genes, such as the Nod factors, can be genetically modified to improve nitrogen-fixing ability, for instance. The same can be done with any other nutrient cycling process [36,307].

Bioremediation: industrialization and current agricultural techniques increase the negative impacts on agricultural land and water by releasing vast amounts of hazardous waste, heavy metals, and organic contaminants, all of which are severe problems, not just for agriculture, but also for human health. Although trace amounts of heavy metals, such as lead (Pb), cadmium (Cd), mercury (Hg), chromium (Cr), zinc (Zn), uranium (Ur), selenium (Se), silver (Ag), gold (Au), nickel (Ni), and arsenic (As) are beneficial to plants, excessive uptake reduces plant growth by interfering with photosynthesis, mineral nutrition, and essential enzyme activities. Industrialization and contemporary farming methods are putting increasing amounts of pressure on the environment. Bioremediation is a process that uses algae, bacteria, fungi, or plants to remove heavy metal ions from a polluted environment. Bioremediation with microorganisms is long-term and sustainable since it helps to restore the natural state of the damaged environment while being cost-effective. Heavy metal detoxification by microorganisms can occur spontaneously, by the addition of native microbial strains or through genetic manipulation. To reduce the active concentration of metal ions present in polluted environments, microorganisms use biosorption, adsorption, compartmentalization of heavy metals into intracellular molecules, metal binding, vacuolar compartmentalization, extracellular mobilization, or immobilization of metal ions [307,308]

Growth and development: microorganisms use a variety of processes to enhance plant development and growth in both normal and stressful settings, including nitrogenase enzyme activity, nitrate reductase activity, siderophore generation, and phytohormone synthesis. Major plant hormones include auxin, cytokinin, gibberellin, abscisic acid, and ethylene, with more phytohormones being discovered. Phytohormones are produced by a variety of microbial species, and they are frequently used in agriculture to improve plant growth and stress tolerance. Plant growth-promoting bacteria (PGPB), also known as rhizobacteria, have been genetically modified to increase the synthesis of stress-induced hormones, antibiotics, antifreeze proteins, trehalose, and lytic enzymes, all of which help plants develop and cope with stress. In order to compete with the already-adapted indigenous microorganisms, PGPR must develop and sustain a biologically active population. Genes that promote growth have been shown to improve strains. As a result, efforts have been undertaken to vary the timing or level of their expression, as well as transfer and express them in different hosts, in order to improve plant growth and development [307].

Genes involved in growth promotion were shown to be effective tools for strain improvements by altering their expression timing and level, or by transferring and expressing them in different hosts to increase plant growth and fitness. Microorganisms modified through genetic engineering have improved specific characteristics, such as the ability to degrade a wide range of contaminants for bioremediation of soil, water, and activated sludge, improved plant biotic and abiotic stress tolerance, and increased phytohormone production, among other things. In a hostile environment, the modified strain can survive and remain active [13]

Stress management: as sessile organisms, plants are subject to abiotic and biotic stress. Diseases, drought, submergence, metal toxicity, salinity, and various other stressors are faced by crops throughout the seasons. Microbes are key regulators of stress through the various biomolecules that are exuded in the form of antibiotics and hormones. The chemical exudates from the microbes activate ISR and induce the resistance mechanism in plants. Genes, such as chitinases and glucanases, have been effectively used to engineer both crops and microbes to enhance the expression of these genes in planta or in microbes for enhanced resistance towards pathogens [309].

While the wild type and mutant microbes have the general functions as stated above, the transition from laboratory to field and market is slow. Transition to market would require the optimization of concoction, determination of concentration, frequency of application, and selection of carriers for these organisms. All of this requires time and funding to fine-tune. While some microbial concoctions have made their way into the market as biofertilizers, biocontrols, soil amendments, and biostimulants, many are still in the laboratory and greenhouse phase, working on optimization. Another factor that has impeded the transition to the utilization of microbes is the efficacy of these compounds compared to chemicals agents, as chemical have wider spectrums of efficacy and and result in more consistent effects on plants and detrimental microbes.

## 7. Future Prospects and Challenges in Plant–Microbe Interactions

Most current and past studies have either selected one of more of the methods employed above to determine the soil microbial structure, density, and function. However, while these techniques do provide some insight on the plant–microbial interactions, they by no means provide a complete picture of the microbial interactions that occur in reality between the plant and microbes in a variety of conditions. Further, rather than identifying the microbes that are present in a particular environment, it would be beneficial for us to know the roles that they play in the environment, singly and in combination with others. Therefore, based on the above listed interactions, it is necessary to “put the pieces together” based on: (1) The microbes present aboveground; (2) The microbes present belowground; (3) How the belowground microbes affect the host and interaction aboveground; (4) The processes and interactions between the host and microbe and root and root; (5) The exudates produced by the plants, the microbes, and their functions; (6) How these affect a plant’s gene expression and immune system; (7) How these microbes affect plant growth, development, and immunity; and (8) Whether there are specific chemical compounds involved in microbe recruitment (and many more).

The above-mentioned information is a consequence of direct or indirect effects of microbes on the host. In recent decades, with the arrival of next generation sequencing platforms, we were able to observe these interactions at the molecular level. The depth of information made available from genomic, proteomics, transcriptomics, and metabolomics has shed some light on the intricacies of the plant–microbe interaction, enabling us to understand the process of disease development, growth and development, immune response, nutrient cycling and absorption, disease suppression, and others. Most of the studies have been directed towards identifying dominant taxa in a particular environment, the effects of plants and microbe exudates on the recruitment of microbes, and the structural architectures of the diversity and communities. There is still much that needs to be studied on the mechanism of recruitment, on the communities influence the plants and each other. Little is known on how the microbial factors influence root exudation and architecture. This information may be manipulated to optimize the microbial communities in the soil and improve overall performance of plants. The following are some applications of metagenome studies conducted in plant–microbe interactions.

(1) Identified productive microbiomes by creating conducive environments for the rhizosphere microbiome to communicate with the plant and surrounding environment.
(2) Applied comparative genomics and metabolomics studies to identify specific rhizobacteria that were naturally selected based on root exudates; optimized utilization of these cultures to increase growth and development.
(3) Identified microbes and their proteomes, able to trigger ISR and SAR across monocots and dicots.
(4) Applied transcriptome profiling to identify defense-associated transcripts involved in innate immunity and plant resistance scenarios.
(5) Identified microbes used in seeding of disease suppressive soil to enhance plant fitness and productivity.
(6) Identified plant-associated microbiomes that influenced different plant traits including abiotic stress tolerance, flowering, growth, and disease suppression. Host co-evolution with the microbiome could be utilized in future crop breeding strategies for low-input sustainable agriculture.
(7) Mapped microbiomes in the soil through all developmental stages, the differences in the proteins exuded. This information may be used to generate microbial concoctions for soil amendments to support growth and yield in all stages.
(8) Exploited beneficial microorganisms and identified emerging pathogens.

With further research and more information being provided from omics-based studies, we expected that more clarity will be obtained concerning plant–microbe interactions.

## Figures and Tables

**Table 1:** The beneficial chemicals exuded by microbes and the benefit to plant defense mechanism.

Plant	Producing Microbes	Beneficial Chemicals	Benefit in Plant Mechanism	Reference
Banana	*Bacillus* spp.	Siderophores	Can inhibit the spread of plant pathogens	[170,171]
**Volatile Organic Compounds (VOCs)**				
*Arabidopsis*	*Bacillus subtilis* GB03 *and Bacillus amyloliquefaciens* IN937a	2,3-Butanediol (2,3-BD)	Enhances Arabidopsis development Elicits ISR towards pathogenic *Erwinia* using ethylene signaling pathways	[172]
Corn and Tobacco	*Bacillus cereus C1L*	Dimethyl disulfide	Protects plants against *Cochliobolus heterostrophus* and *Botrytis cinerea*	[173,174]
*Arabidopsis thaliana*	*Trichoderma asperellum*	6-pentyl-pyrone	Boosts plant defense responses while suppressing *B. cinerea* and *A. alternata* sporulation	[175]
**Phytohormones**				
*Medicago truncatula*	*Salmonella*	Auxins	Aids in the development of new organs Inhibits plant defenses Controls phytobacteria pathogenicity	[176,177]
*Oryza sativa*	*Bacillus amyloliquefaciens*	Abscisic acid (ABA)	Improves resistance towards salinity Integrates signaling during stress exposure	[178]
*Arabidopsis thaliana*	*Pseudomonas fluorescens*	Cytokinin	Maintains proliferation and differentiation of the cell Inhibits premature leaf senescence	[179]
Various plant	*Azotobacter*, *Azospirillum*, *Pseudomonas*, *Azotobacter*, *Burkholderia*, *Bacillus*	Gibberellins	Important in dormancy of floral organ development, lateral shoot growth	[180]
Tomato	*Fusarium oxysporum*	Jasmonic acid (JA)	Plant defenses against necrotrophic pathogens Activated ISR	[125,181]
*Metasequoia glyptostroboides*, *Ginkgo biloba, Taxus brevifolia,* etc.	*Pseudomonas tremae* *Curtobacterium herbarum*	Salicylic acid (SA)	Crucial role in plant stress tolerance Increases resistance from tobacco wildfire disease	[182,183]
Apple	*Pseudomonas syringae*	Ethylene	Promotes fruit ripening	[184]
**Signaling Molecules**				
*Piloderma–Pinus*, orchids, etc.	Mycorrhizal fungi	Small signaling proteins (SSPs)	Function as mutualistic effectors that promote mycorrhization in their plant hosts	[185,186]
*Arabidopsis thaliana*	PGPR	*N*-acyl-homoserine lactones (AHL)	Stimulate the plant’s host to use strategies to counteract the bacterial signals Helps with root development, plant defense, stress response, hormonal balance, and metabolism	[187,188,189,190,191]
Grapevine, lettuce, etc.	*Pseudomonas* sp., *Burkholderia* sp., and *Bacillus* sp.	Rhamnolipids and Lipopeptides	Plant immunity to phytopathogens is increased when the immune system of the plant is stimulated	[192]
Tomato	Pseudomonas fluorescens	2,4-Diacetylphloroglucinol (DAPG)	Promotes root development via auxin dependent signaling pathway	[193]
*Arabidopsis*	*Pseudomonas aeruginosa*	Pyocyanin	Promotes ISR root development	[194]
*Arabidopsis*	Nematodes	Ascaroside pheromones	Triggers the production of defense genes and microbial infections tolerance	[195]
**Enzymes**				
Various plant	*Trichoderma harzianum, Trichoderma virens and Trichoderma viride*	Lytic enzymes	Eradicate plant disease-causing substances in plants; inhibits a wide range of fungal phytopathogens that arise from the air or soil	[196]
*Doryanthes excelsa* *Protea montana*	*Proteobacteria* and *Acidobacteria*	Phosphatase	Mineralize organic P compounds. Control the carbon sink’s capacity, given that P uptake may be a limiting factor, in continual growth, in changing climates	[197]
*Phaseolus vulgaris*	*Rhizobium etli*	Trehalose	Increases the number of root nodules and enhances N_2_ fixation Acts as an osmoprotectant	[198,199]
Canola	*Trichoderma atroviride*	Chitinase	Resistance against the stem rot disease caused by *Sclerotinia sclerotiorum* Attacks the chitin of the pathogenic fungi	[200,201]
Rice	*Bacillus* spp.	Proteases	Boost antioxidant defense functions Reduce oxidative damage and occurrence of blast disease	[202]

## Data Availability

Not applicable.

## Supplementary Materials

The following are available online at https://www.mdpi.com/article/10.3390/ijms221910388/s1.
