# Supplementary material for: Plant–Microbe Interaction: Aboveground to Belowground, from the Good to the Bad
Source: Int J Mol Sci. 2021 Sep 27;22(19):10388. doi: 10.3390/ijms221910388 (PMC8508622; doi:10.3390/ijms221910388)
Supplement: Supplementary file 1 [file ijms-22-10388-s001.zip › ijms-1336863-supplementary.pdf]

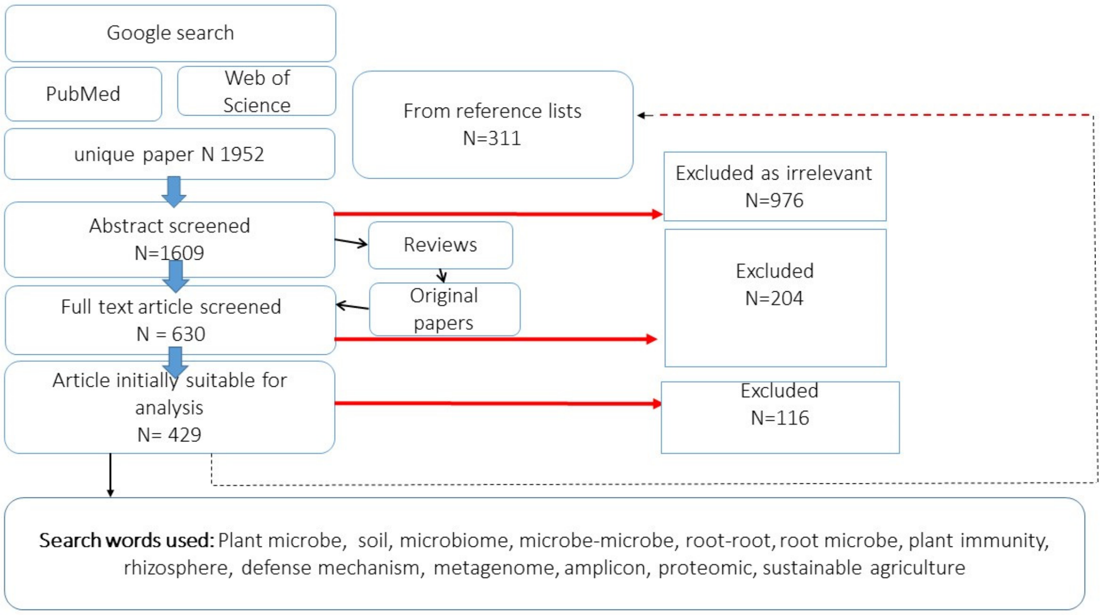

Figure S1. This is a diagrammatic representation of how the papers were selected for the Systematic Review on Plant Microbe Interactions. The above three search engines were deployed and first the search was conducted for paper released in this area. From the titles that fit into the main search item, selected papers were identified as most suitable for further abstract screening. Abstracts that were within the framework of the paper were then used to obtain full papers. A selection of about two thirds of this papers were made for survey of content. From the further papers were excluded. The manuscript was finally written with 309 papers that were either reviews, research articles or book chapters.
